# DArT, SNP, and SSR analyses of genetic diversity in *Lolium perenne* L. using bulk sampling

**DOI:** 10.1186/s12863-017-0589-0

**Published:** 2018-01-22

**Authors:** Siyang Liu, Ulf Feuerstein, Wilbert Luesink, Sabine Schulze, Torben Asp, Bruno Studer, Heiko C. Becker, Klaus J. Dehmer

**Affiliations:** 1 Leibniz Institute of Plant Genetics and Crop Plant Research (IPK), Corrensstrasse 3, 06466 Gatersleben, Germany; 2 Deutsche Saatgutveredelung KG, Weissenburger Strasse 5, 59557 Lippstadt, Germany; 3 Norddeutsche Pflanzenzucht Hans-Georg Lembke KG, Inselstrasse 15, 23999 Malchow/Poel, Germany; 4 Saatzucht Steinach GmbH & Co KG, Wittelsbacherstrasse 15, 94377 Steinach, Germany; 5 Institute of Genetics and Biotechnology, Faculty of Science and Technology, Aarhus University, Forsøgsvej 1, 4200 Slaglese, Denmark; 6 Molecular Plant Breeding, Institute of Agricultural Sciences, ETH Zurich, Universitätstrasse 2, 8092 Zurich, Switzerland; 7 Georg-August-University Göttingen, Von Siebold-Strasse 8, 37075 Göttingen, Germany

**Keywords:** Perennial ryegrass, Genetic diversity, Genetic resources, Genetic pools, Genotyping, Hybrid breeding

## Abstract

**Background:**

*Lolium perenne* L. is the most important forage grass species in temperate regions. It is also considered as a sustainable source of biomass for energy production. However, improvement in biomass yield has been limited by comparison with other major crops. More efficient utilisation of genetic resources and improved breeding schemes are required to advance *L. perenne* breeding. In an attempt to elucidate the extent of genetic diversity in *L. perenne*, 1384 DArT, 182 SNP and 48 SSR markers were applied to 297 accessions (Set I) contributed by three German breeding companies and the IPK Genebank. Due to the heterogeneous nature of *Lolium* accessions, bulk samples were used. Apart from germplasm set I, additional set II and set III was used to determine the reproducibility of marker system and judge the feasibility of bulk strategy in this study.

**Results:**

By assessing different bulk sizes, 24 individuals per sample were shown to be a representative number of plants to discriminate different accessions. Among the 297 accessions, all marker types revealed a high polymorphism rate; 1.99, 2.00 and 8.19 alleles, were obtained per locus on average using DArTs, SNPs and SSRs, respectively. The Jaccard distance for DArT markers ranged from 0.00 to 0.73, the Modified Roger’s distance (MRD) for SNP markers ranged from 0.03 to 0.52, and for SSR markers from 0.26 to 0.76. Gene diversity for dominant DArT and co-dominant SNP and SSR markers was found to be 0.26, 0.32 and 0.45, respectively. DArT markers showed the highest consistency and reproducibility.

**Conclusion:**

The resulting data were evaluated using a number of different classification methods, but none of the methods showed a clear differentiation into distinct genetic pools. With regard to hybrid breeding, this will possibly impede substantial progress towards increased biomass yields of *L. perenne* by utilising heterosis.

**Electronic supplementary material:**

The online version of this article (10.1186/s12863-017-0589-0) contains supplementary material, which is available to authorized users.

## Background

*Lolium*, which shares evolutionary lineage with economically important cereal crops like rice (*Oryza sativa*), wheat (*Triticum aestivum*) and barley (*Hordeum vulgare*) [1], is generally considered the major forage grass genus in temperate regions like Northwest and Central Europe, Australia, New Zealand, parts of Japan, South Africa and South America [2]. *Lolium perenne* L. is the most important *Lolium* species in terms of the number of registered varieties and global seed production [3]. It is an outcrossing naturally diploid species (2n = 2× = 14). Apart from its economic importance in fodder production, *L. perenne* also serves as turf grass or amenity grass [4]. Compared with other *Lolium* species like *L. multiflorum* (Italian ryegrass) and *L. × hybridum* (hybrid ryegrass), *L. perenne* displays greater persistence and digestibility [3]. Additionally, some genotypes possess strong resistance against biotic and abiotic stresses [5] and have high yield potential. Therefore it has been also proposed as a candidate plant for biogas production [6, 7].

Breeding of *L. perenne* can be traced back to the 1920s [8]. Major breeding achievements include improvements in yield and persistence, and increases in nutritional value [2], as well as the induction of tetraploidy by treatment with colchicine [9]. However, the gain in yield is not comparable to that in cereal crops [10, 11]. To advance the yield improvement, more efficient utilisation of plant genetic resources is required. Polymorphic molecular markers could provide reliable characterisation of germplasm resources [12] and therefore offer possibilities to identify germplasm structure or even heterotic patterns [13].

To date, the diversity of various *L. perenne* germplasm or cultivars has been examined with different molecular marker types including AFLP [14–16], ISSR [17–19], RAPD [8, 20], and SSR [21, 22]. However, the number of examined accessions in these studies was generally limited. This can be partially attributed to the large within population variability [15, 16, 20] which indicates that multiple individuals have to be genotyped to be representative for a certain accession. Bulk sampling is an alternative that can allow the investigation of increased numbers of accessions. Guthridge et al. [15] studied six *L. perenne* populations with a bulk sampling strategy and AFLP markers. They found that mutual relationships revealed by bulk sampling were consistent with the results obtained by individual analysis. By applying bulked samples, Nestmann et al. [23] investigated the influence of differing grassland composition on the differentiation of *L. perenne* populations with the SNP markers developed by Sretenovic Rajicic et al. [24].Byrne et al. [25] used Genotyping-by-Sequencing (GBS) to analyse populations. With their new approach of “Genome Wide Allele Frequency Fingerprints” (GWAFFs) they were able to distinguish between eight *L. perenne* cultivars. However, a diversity study in *L. perenne* for large germplasm sets using bulked samples to our knowledge has not yet been published.

The objectives of this study were a) to assess the feasibility of bulk sampling for diversity studies of a large germplasm set of *L. perenne*; b) to examine the underlying population patterns and gene diversity within the collections; c) to compare the performance of different marker types in analysing bulked ryegrass samples. In this study, DArT, SNP and SSR markers were chosen. They have common features, such as the availability of automated platforms, but also have several differences. For DArTs, the detection of polymorphisms is not dependent on prior knowledge of DNA sequence [26], therefore they are particularly ideal for species with limited genome information. However, their inherent dominant nature reduces the information content [27, 28]. SNPs and SSRs are both co-dominant marker types and are highly polymorphic, but currently publicly available primers and sequences are limited.

## Methods

### Plant material

A set of 297 accessions of *L. perenne* including varieties, breeding materials and ecotypes was compiled. Donors of materials included the three German breeding companies Deutsche Saatveredelung KG (DSV), Norddeutsche Pflanzenzucht Hans-Georg Lembke KG (NPZ), and Saatzucht Steinach GmbH & Co KG (SZS), Leibniz Institute of Plant Genetics and Crop Plant Research, IPK. The ploidy status and geographical origin are available for the majority of the accessions (Table 1; Additional file 1: Table S1). It is expected that this material represents a broad range of variation that exists within the German *L. perenne* breeding pool as a whole. We denote these 297 accessions as Set I. In addition to Set I, six *L. perenne* accessions from Set I and one *L. multiflorum* accession were replicated in order to test the reproducibility of the marker systems. These eight samples were denominated as Set II. In order to assess the influence of bulk size, various sampling strategies including one, 12, 24, 48 and 100 individuals per bulk were applied to four *L. perenne* accessions from Set I: GR2725 (13 samples), GR2915 (nine samples), GR3107 (eight samples), and GR8502 (seven samples). We denoted these 37 samples as Set III (Table 2). In total, Set I, Set II, and Set III consisted of 342 samples.

**Table 1:** Summary of Set I containing 297 *L. perenne* accessions classified by geographical origin, ploidy level, biological status and donors

Geographical Origin^a^			
Western EU	197	Eastern EU	10
Northern EU	37	Oceania	5
Southern EU	5	Unknown	43
Ploidy Level			
Diploid	232	Tetraploid	65
Biological Status			
Breeding		Ecotype	43
Material	206	Landrace	2
Variety	42	Unknown	4
Donor			
DSV	126	IPK	48
NPZ	90	SZS	27
Other^b^	6		

^a^the classification refers to United Nations Statistics Division. EU: Europe

^b^standard cultivars were not assigned to any particular contributor

**Table 2:** Summary of Set III containing 4 selected genebank (GR2725, GR2915, GR3107 and GR8502) accessions and differed bulk size

Bulk size	GR2725	GR2915	GR3107	GR8502
1 individual/bulk	1	1	1	1
12 individuals/bulk	4	4	3	2
24 individuals/bulk	4	2	2	2
33 individuals/bulk	2	–	–	–
48 individuals/bulk	1	1	1	1
100 individuals/bulk	1	1	1	1
total sample per accession	13	9	8	7
*Total*	37			

The number in the table indicates the number of samples per bulk size and accession combination

Seeds of all accessions were sown and young leaves were sampled after four weeks. Leaf tissue was punched out with a metal rod (ø 1.8 mm) from the upper half of the leaf lamina. For Set I and Set II, leaves from 30 individuals per accession were equally pooled for each sample to obtain approximately 100 mg of fresh leaf material following Nestmann et al. [23]. For some accessions from NPZ, samples are only taken from clones (Additional file 1: Table S1).

### Molecular markers

The 342 samples were genotyped with DArT, SNP and SSR markers. DNA from samples of Sets I and II was initially extracted at Saaten-Union Biotech GmbH (Leopoldshöhe, Germany) where the SSR markers were subsequently amplified. DNA from Set III was extracted at IPK. All prepared DNA samples were also distributed to TraitGenetics GmbH (Gatersleben, Germany) for SNP marker genotyping and to Diversity Arrays Technology (Canberra, Australia) for DArT marker genotyping. Pre-selection of markers was performed by the corresponding company.

DArT markers were scored for presence/absence. In general, only markers meeting a threshold of 80 (*p* value) were included in the analyses. Yet additional markers with high call and low discordance rates were also included according to recommendations by the service provider (mean p value 73.5%, minimum 46.8%, maximum 93.9%). Among all the DArT markers applied in this study, 114 were mapped [29], consisting of 18, 17, 16, 21, 22, 11, and nine markers on Chromosomes 1 to 7, respectively. The location of the rest of the DArT markers was unknown.

For the SNP markers, sequences were taken from Studer et al. [30], and frequencies for the major allele were directly inferred via Illumina GoldenGate genotyping assays. For each locus and each sample, the relative signal intensity scanned by a slide reader were converted into frequency data via the GenomeStudio software (accuracy according to the service provider +/− 10%). Based on genetic maps (University of Aarhus and IPK Gatersleben, personal communication K.J. Dehmer), 23, 22, 29, 38, 17, 20, 27 markers were located on linkage groups 1 to 7, the locations of the remaining six markers were unknown.

For the SSR markers, primer combinations recommended by the service provider were employed. The electrophoresis patterns were recorded followed by a manual scoring of the peaks. Weak peaks were not included due to their presumably low influence on the allelic frequencies at a locus. A total of 48 marker loci were examined. The initial presence/absence scoring of the alleles was standardized by attributing a weight to each allele in order to take into account the different number of alleles per locus. By this, an overestimation of loci with multiple alleles in comparison to loci with only two alleles could be avoided. Thus, scores for every allele from a pentaallelic-locus was e.g. multiplied by a factor of 0.2, whereas data for each allele from a biallelic locus was multiplied by 0.5.To assign the linkage groups, five to 12 markers were used on linkage groups 1 to 7 according to the maps constructed by Studer et al. [31].

Marker loci containing 30% or more missing values across all samples were excluded from the dataset leading to a final marker dataset containing 1384 DArT, 182 SNP and 48 SSR marker loci with average missing value rates of 5.2%, 3.3% and 3.2% respectively.

### Statistical analysis

Genetic distances (GD) were calculated for all samples. Due to the differences between marker types, different distance measures were calculated following Reif et al. [27]. Distances of co-dominant SNP and SSR markers were assessed using the Modified Roger’s Distance (MRD) [32] whereas dominant DArT markers were calculated using the Jaccard Distance (JD) [33]. Pearson correlation coefficients were determined between all pairs of distances for the three marker types. The mean values of GD for Set I as well as the replicated samples in Set II were also calculated. Nei’s gene diversity [34], mathematically equal to the polymorphic information content (PIC) [35], was calculated for each locus separately for Set I.

Based on the GD matrices, phenograms of the 342 samples were constructed with Unweighted Pair Group Method with Arithmetic means (UPGMA) to check the applicability of the bulk sampling strategy. The cophenetic index was calculated as a pairwise Pearson correlation coefficient between the cophenetic matrix and genetic distance matrix to check the fitness of the constructed phenogram.

Cluster analysis was conducted for DArT markers using the software STRUCTURE [36]. Potential subgroups (K = 1–10) were tested with five replicates each by applying an admixture model. The burn-in time and number of iterations were both set to 100,000. The ad hoc criterion was utilised to determine the most likely group numbers [37]. However, the allelic frequency data from SNP and SSR markers were not executable in STRUCTURE. To have a comparable platform which is applicable to all marker types, PCo (Principal Coordinate) based clustering was conducted using principal coordinates (PCo) 1–100 calculated from the corresponding GD matrices to examine the sub-groups using all three marker types [38]. The number of potential clusters was set as 1–20 in this analysis.

Analysis of Molecular Variance (AMOVA) based on GD was implemented for Set I with different categories defined by donor, geographical origin, ploidy level and biological status (Table 1). Accessions containing incomplete information were discarded. AMOVA was also applied to the replicate samples in Set II in order to dissect the proportion of the variance within and among replications to compare the marker types.

To simplify the multivariate dataset and visualise the population patterns, Principal Coordinate Analysis (PCoA) was conducted with two dimensions for three marker types based on their corresponding GD matrices. PCoA was plotted for Set I to present the variation within the germplasm. Additionally, a PCoA plot was also constructed for Set III to inspect the variation caused by various bulk sizes (Table 2).

Bootstrapping analysis was carried out to detect the variance generated by resampling subsets of complete marker data sets and to determine the minimum marker number required to achieve the acceptable accuracy as the complete data set. Of the total marker numbers, 2, 4, 10, 15, 20, 25, 30, 40, 50, 60, 70, 80 and 90% were randomly selected with 100 repetitions each to form subsets of the entire data set. MRD or JD was further calculated for each of the subsets. The Coefficient of Variation (CV) across replications was determined because CV is not influenced by data with different mean values and is more suitable for comparisons between different marker types [13].

The R platform was used for all calculations, simulations and graphics [39]. Specially, PCo-based clustering was conducted with the R package mclust [38]. Graphics were prepared by R built-in graphic functions and the package ggplot2 [40].

## Results

### Genetic diversity within the germplasm set I

**Fig. 1:**
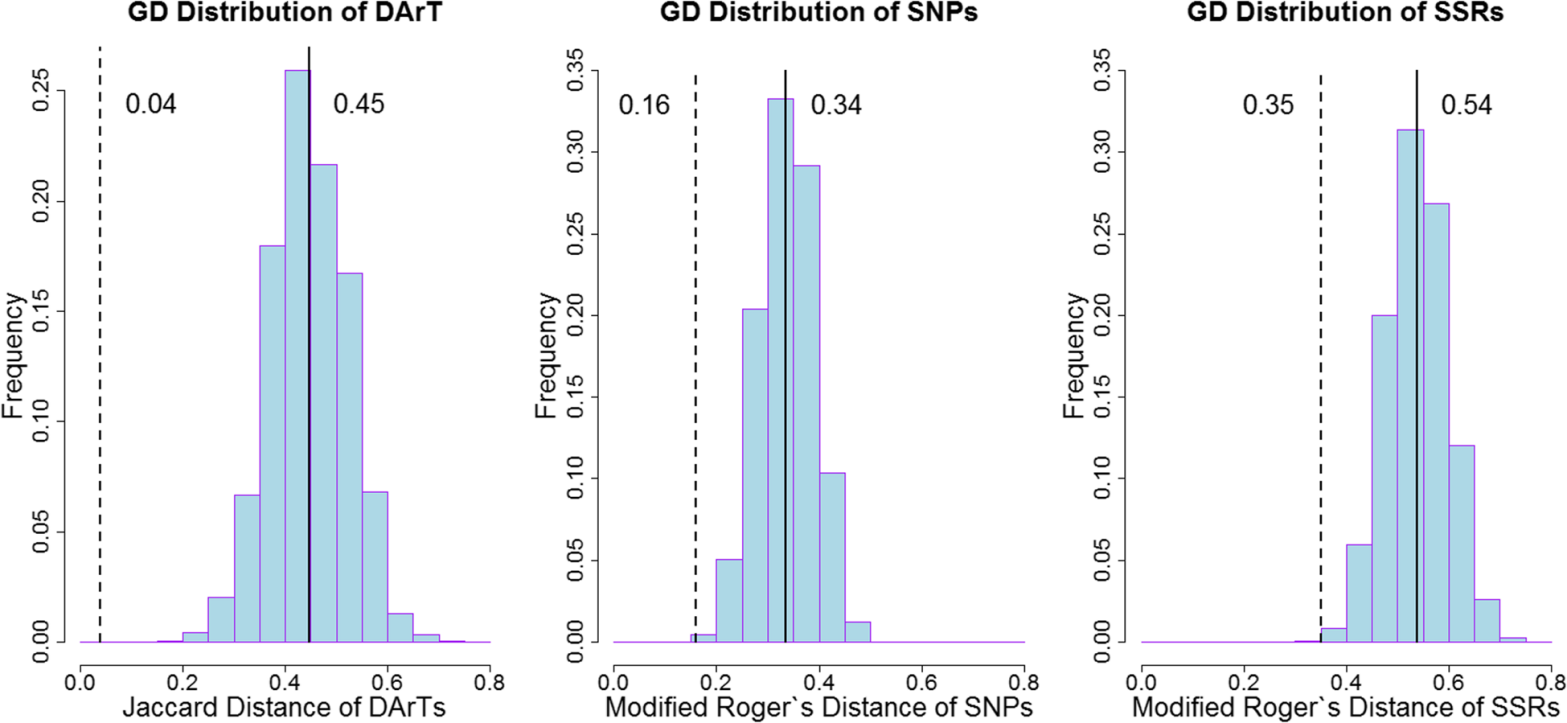
Distribution of genetic distances (GD) obtained using DArT, SNP and SSR* markers for 297 *L. perenne* accessions (Set I). Solid line indicates mean value of the corresponding genetic distance of 297 accessions. Dashed line indicates mean value of replicates in Set II. *: for SSRs, one replicate sample was excluded due to a high missing value rate

A total of 342 bulk samples were genotyped using DArT, SNP and SSR markers, the corresponding genetic distances were calculated for all samples and Nei’s gene diversity was estimated for Set I using various categories. For Set I, 1380 of the 1384 DArT marker loci were polymorphic; all 182 SNP marker loci were polymorphic; the number of alleles for SSR ranged from two to 23 with an average of 8.2 alleles per locus. The JD for DArT markers ranged from 0.00 to 0.73 resulting in a mean distance of 0.45. For SNPs, the MRD was between 0.03 and 0.52 with an average value of 0.34; for SSRs, the MRD ranged from 0.26 to 0.76 with a mean value of 0.54. The distribution of the distance estimates for the three marker types are plotted in Fig. 1. In Set II, pairwise genetic distances within the replicated samples were also calculated to estimate the reproducibility of the different genotyping systems. JDs between replications based on DArT markers were 0.04 on average, while the mean of the MRDs based on SNP markers and SSR markers were 0.16 and 0.34, respectively (Fig. 1). For all 342 samples, the correlation coefficients of GD estimates were 0.83*** between DArTs and SNPs, 0.68*** between DArTs and SSR, 0.70*** between SSRs and SNPs. Nei’s gene diversity for Set I based on DArTs, SNPs and SSRs was 0.26, 0.32 and 0.54, respectively.

Three indices, namely unique alleles, average alleles per locus as well as gene diversity based on groups classified in passport data were calculated (Table 3). Considering geographic origin, we observed higher numbers of unique alleles, average alleles per locus as well as gene diversity for material from Western and Northern Europe and this holds true for all three marker types. Considering ploidy levels, diploid material showed more unique alleles with SSRs and DArTs, but gene diversity was similar. Breeding material, ecotypes and material from DSV and NPZ showed more unique alleles as well as average alleles per locus with DArT and SSR markers than with other groups; with SNPs, however, no distinct differences could be identified.

**Table 3:** Number of unique alleles, average alleles per locus and gene diversity based on DArT, SNP and SSR markers for 297 *Lolium perenne* accessions (Set I) classified by geographical origin, ploidy level, biological status and donors

			DArT			SNP			SSR		
		Number of accessions^a^	Unique alleles	Average alleles per locus	Gene diversity	Unique alleles^b^	Average alleles per locus	Gene diversity	Unique alleles	Average alleles per locus	Gene diversity
Geographical Origin	Eastern EU ^c^	10	1	1.63	0.21	0	2.00	0.31	1	3.29	0.47
	Northern EU	37	1	1.89	0.25	0	2.00	0.31	11	5.50	0.53
	Oceania	5	0	1.52	0.21	0	1.99	0.3	4	3.17	0.46
	Southern EU	5	0	1.56	0.22	0	1.97	0.29	1	3.00	0.48
	Western EU	197	23	1.99	0.26	0	2.00	0.32	60	7.44	0.54
Ploidy Level	2×	232	86	1.99	0.26	0	2.00	0.31	118	8.00	0.54
	4×	65	6	1.99	0.25	0	2.00	0.32	9	5.69	0.53
Biological Status	Breeding Material	206	18	1.99	0.26	0	2.00	0.32	64	7.29	0.55
	Variety	42	1	1.88	0.22	0	2.00	0.31	8	5.25	0.51
	Ecotypes	45	2	1.91	0.26	0	2.00	0.32	25	5.92	0.54
Donor	DSV	126	13	1.98	0.26	0	2.00	0.32	34	6.94	0.54
	IPK	48	2	1.88	0.22	0	2.00	0.31	13	5.27	0.51
	NPZ	90	3	1.94	0.27	0	2.00	0.32	26	6.54	0.55
	SZS	27	1	1.83	0.23	0	2.00	0.31	4	4.69	0.53

^a^unknown material was excluded from the summary

^b^based on the standard that the specific group contains alleles with frequency higher than 0 while in the rest of material the allelic frequency is 0

^c^EU: Europe

### Feasibility of bulk sampling

**Fig. 2:**
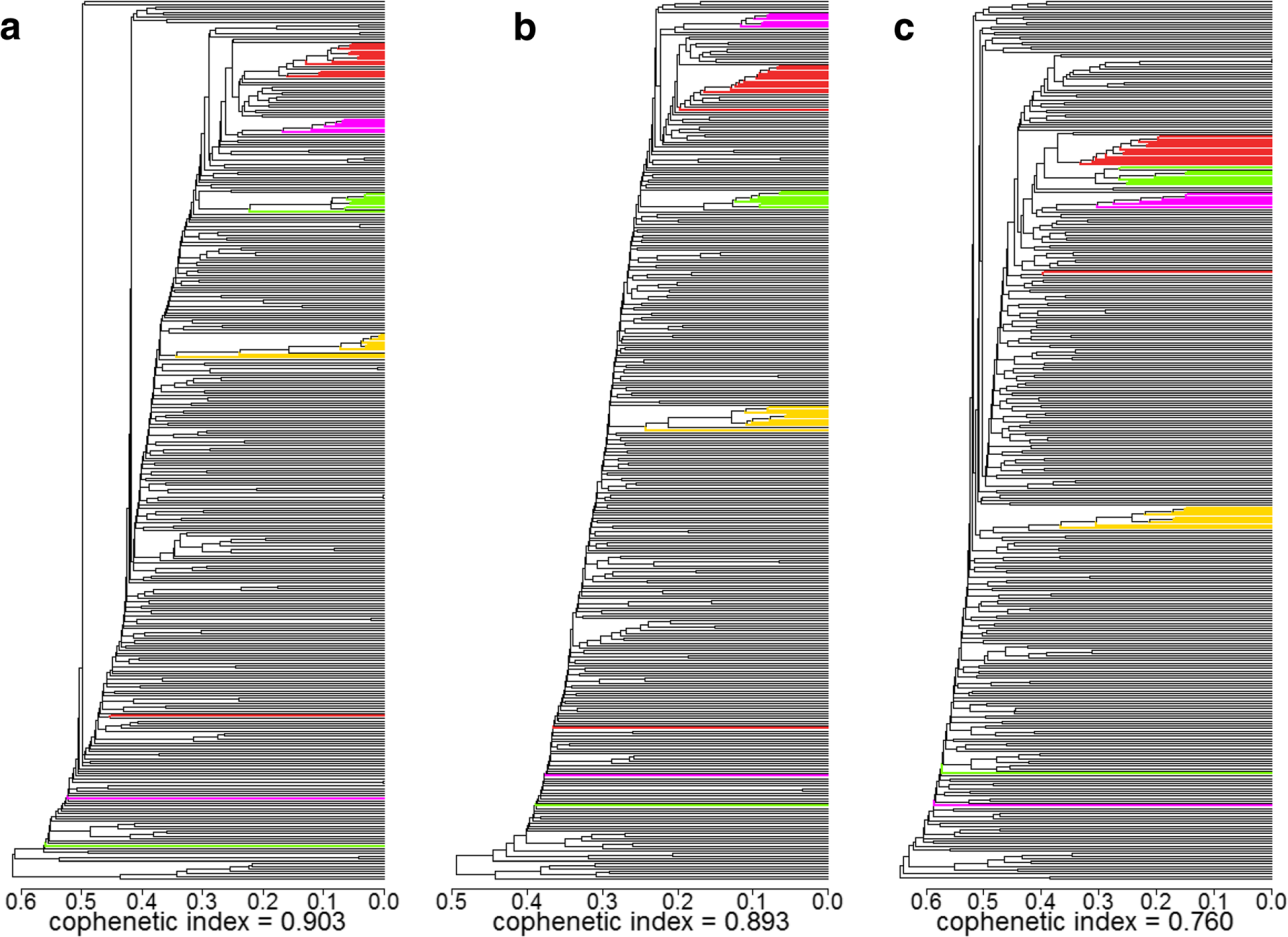
Phenograms based on genetic distances obtained using **a**) DArT, **b**) SNP and **c**) SSR markers for 297 *L. perenne* (Set I), eight repeats (set II) and 37 re-bulked samples (Set III, highlighted). red: GR2725; green: GR3107; yellow: GR2915; pink: GR8502

Phenograms based on DArT, SNP and SSR markers were constructed for all 342 samples. Set III was highlighted with four different colours (Fig. 2). For all marker types, four groups were formed, which is in accordance with their corresponding accessions. Regardless of the different marker types, some samples were clustered outside of their expected group, and all of them belong to the 1 individual/bulk sample group summarised in Table 2. Cophenetic indices for the three phenograms were 0.90, 0.90 and 0.76, respectively, indicating an almost ideal representation of the information contained in the GD matrices.

**Fig. 3:**
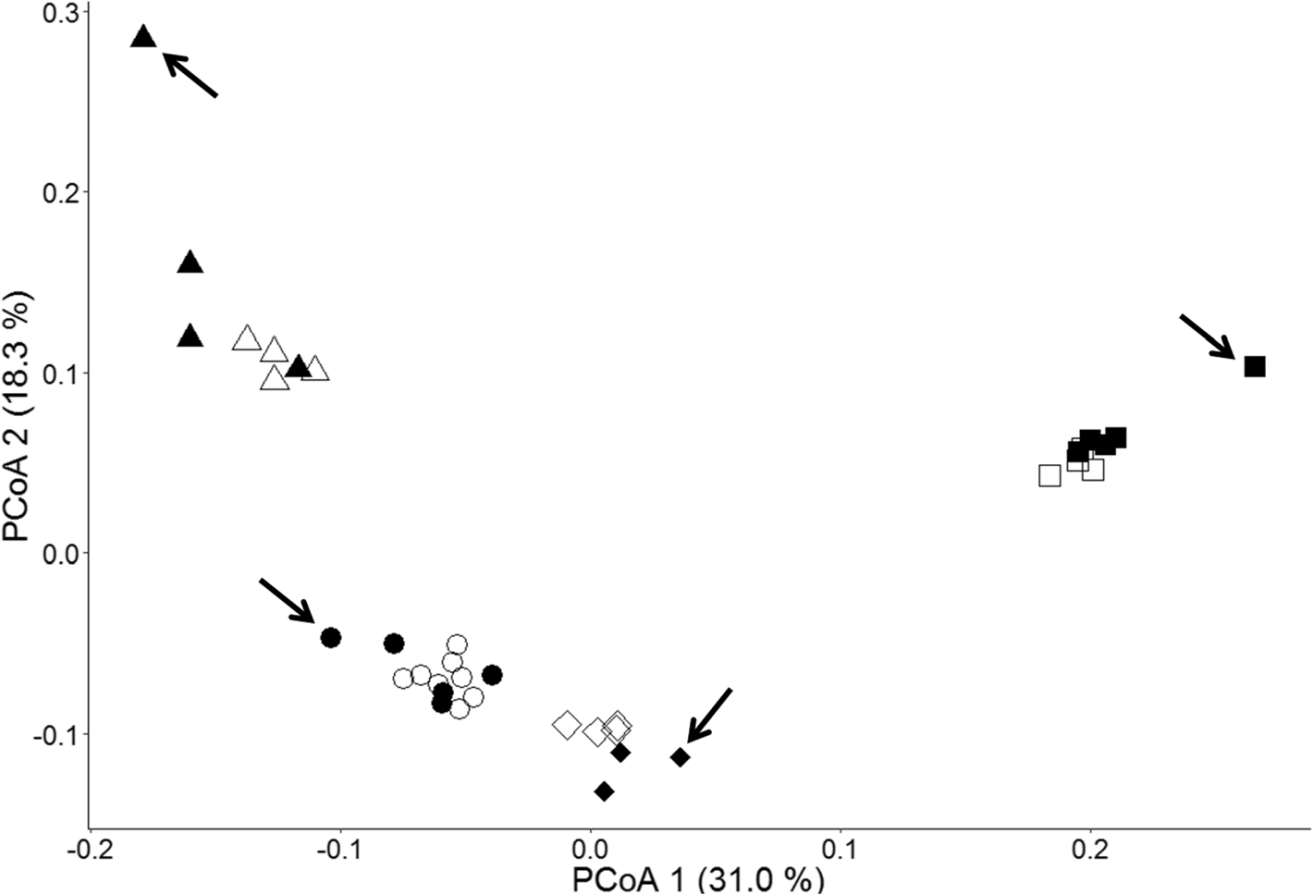
PCoA (Principal coordinate analysis) plot of Set III containing four genebank accessions (GR2725, GR3107, GR2915 and GR8502) of various bulk sizes to illustrate the influence of bulk size. Based on DArT marker. Shapes: circle: GR2725; triangle: GR3107; square: GR2915; diamond: GR8502; empty: bulk size equals to or above 24 ind./sample; filled: bulk size below 24 ind./sample; arrows: samples containing only one individual

PCoA analyses on Set III with DArT markers separating the small bulk size samples (sample containing less than 24 individuals) and large bulk size samples (samples containing 24 or more individuals) revealed greater consistency for the samples with larger bulk sizes (Fig. 3). Similar with the phenograms, four groups could be clearly defined and samples containing only one individual displayed the highest variability. The patterns of SNP and SSR markers were similar to those of DArT markers (Additional file 2: Figure S1).

### Comparison of marker types

**Fig. 4:**
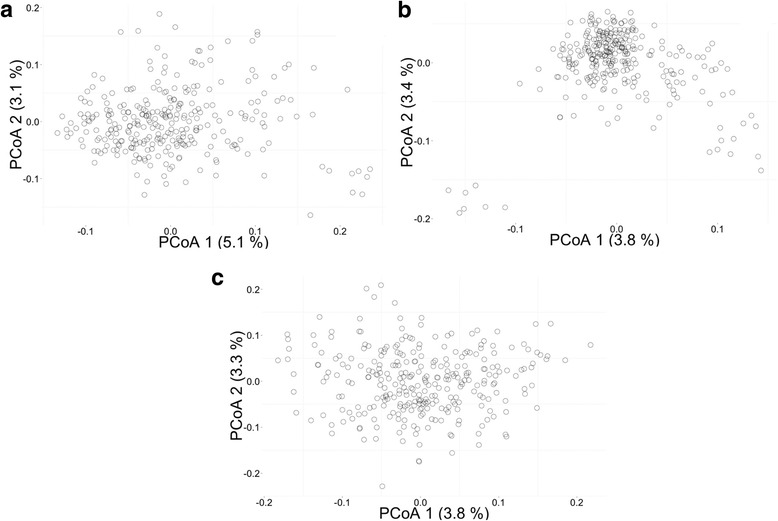
Principal coordinate analysis of germplasm set I including 297 L. per*enne* accessions. (**a**) DArT, (**b**) SNP and (**c**) SSR markers

In PCoA on germplasm sets I, the first two principal coordinates only explained 5.1% and 3.1% of the molecular variance for DArTs, 3.8%, 3.4% for SNPs and 3.8% and 3.3% for SSRs (Fig. 4), which could be explained by the high number of accessions involved and the lack of structure within the material. For all three marker types, large variation could be identified but a clear identification of some potential groups was difficult. In DArTs and SNPs, several material appears to be distinct and all of these accessions are clones from NPZ.

The ad hoc criteria in STRUCTURE analysis for DArTs indicated eight potential clusters in Set I (Additional file 2: Figure S2a). However, none of the categories defined in Table 1 could reasonably separate different clusters. Instead, clusters are dispersedly assigned to different categories (Additional file 2: Figure S2 b,c,d). PCo-based clustering revealed rather variable numbers of clusters within the dataset when a small number of PCos was used for the analyses, the estimated number of clusters tended to be constant when more PCos were considered. For DArTs, the number of clusters ranged from 2 to 16 with PCo 1 to 64 and stabilised at four clusters when PCo 65 or more were considered. For SNPs, the number of groups varied in the range from two to 11 with PCo 1 to 63 and stabilised at three clusters after PCo 64. For SSRs, the stabilisation was reached much earlier than for DArTs and SNPs: after PCo 11, only one cluster was suggested by the model (Additional file 2: Figure S3a). Despite the identification of several clusters after stabilisation for DArTs and SNPs, the majority of the material (over 95%) was assigned to only one group (Additional file 2: Figure S3b, c, d).

In order to identify the potential structures within geographical origin, ploidy levels, biological status or donors, AMOVA was applied to Set I. Though all the factors were significant at *p* = 0.01, only a small proportion of the variance could be explained by the defined factors (Table 4), which is in accordance with the STRUCTURE analysis.

**Table 4:** AMOVA for 297 *Lolium perenne* accessions based on genetic distance using DArT, SNP and SSR markers and extent of variation classified by geographical origin, ploidy level, biological status and donor (for the Complete AMOVA see Additional file 1: Table S2)

	DArTs		SNPs		SSRs	
	Variation [%]		Variation [%]		Variation [%]	
	Among	Within	Among	Within	Among	Within
Geographical Origin	2.64	97.36	1.36	98.64	2.60	97.40
Ploidy Level	2.76	97.23	6.39	93.61	1.97	98.03
Biological Status	2.81	97.19	1.49	98.51	1.88	98.12
Donor	3.83	96.17	1.88	98.11	2.51	97.49

Material with unknown origin and standard cultivars were removed in this analysis; Variance component was all significant at *p* = 0.01 after 1000 permutations

Comparing the mean estimates among pairs of replicates in Set II, DArT markers showed the highest reproducibility. This is also supported by the AMOVA results for the pairwise replications in Set II, where the accessions could explain 99% (DArTs), 70% (SNPs) and 52% (SSRs) of the total variance (Additional file 1: Table S3).

**Fig. 5:**
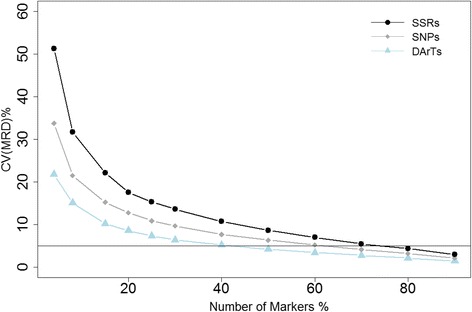
Combined bootstrapping analyses using genetic distances based on DArT, SNP and SSR markers for 297 *L. perenne* accessions (set I). Mean coefficient of variation (CV) of corresponding distance estimates over 100 replications were calculated by resampling a certain percentage of the entire marker dataset. Horizontal line indicates the given threshold of 5% in CV

The combined bootstrapping analysis showed that the CV (coefficient of variation) of the GD estimates among pairs of accessions exponentially decreased when the number of markers selected increased (Fig. 5). As a result, DArTs presented lower CV value than SNPs and the highest CV was always obtained using SSRs. 40% (554), 60% (110) and 75% (36) of the total DArT, SNP and SSR marker sets were able to provide results similar to the complete data set.

## Discussion

### The feasibility of bulk sampling for detection of genetic variation in *L. perenne*

In the current study, a large germplasm set was genotyped using a bulk sampling strategy. Because the ability to provide consistent distinctiveness between accessions is of fundamental importance for diversity and population structure studies, a special set of samples (Set III) was used to inspect the feasibility of the bulk sampling strategy. In the phenograms constructed using the entire sample sets with Set III being highlighted, GR2725, GR2915, GR3107 and GR8502 formed their own distinct clusters (Fig. 2), therefore all three marker systems should be suitable for diversity studies with bulk sampling in *L. perenne*. Our finding is consistent with Guthridge [15] who used AFLP markers and compared their discriminative capability in distinguishing cultivars using multiple individual samples and bulked samples(20 individuals/bulk) and concluded that the results from bulk samples were consistent with those from individual samples.

Besides a suitable genotyping platform, an appropriate sampling size is also essential for the success of bulk sampling strategy. In order to further investigate the effect of the bulk size, we divided each accession in Set III into groups with large number vs. small number of seedlings in the PCoA (Fig. 3). Samples containing one or 12 individuals were defined as small bulk samples and samples containing 24 or more individuals were defined as large bulks. Due to the high level of within population variation [42] it is expected that a large number of individuals is required to represent a certain accession. As expected, a clear trend was identified where bulks with higher sample numbers being more constant than bulks based on fewer individuals. This observation holds true for all three marker types. Similarly, the extremes in the phenograms (Fig. 2) were always observed for samples containing only one individual. These samples could be very different from their corresponding group, as in the case of GR3107. Based on this result, a bulk size of more than 24 individuals should be sufficient for a reliable, bulk-based discrimination of different populations in *L. perenne*. The bulk size of 30 individuals used in Set I was above this threshold and therefore the bulking procedure should be appropriate for our purpose. Related studies have shown that although a small bulk with 3–5 individuals is appropriate for minor allele detection [41](Gilbert et al. 1999), 20–30 individuals per bulk are required for a reliable identification of accessions or cultivars [15, 20]. Our results support these previous studies.

For the SSR markers, 8.4 alleles per locus were found on average, which is lower than the 9.9 [43], 13.3 [22] and 19.4 [21] found in other studies, even though a much higher number of accessions was examined in our study. The limited number of alleles might be attributed to the usage of bulk samples. Unlike in genotyping for an individual sample, multiple peaks for a certain primer pair are possible for bulk samples of ryegrass accessions during SSR data scoring. To more reasonably and accurately estimate allelic frequency, weak peaks in the banding profiles were eliminated. This reduces the ability to detect rare alleles. In addition, bulk sampling is not ideal for rare allele identification due to the sensitivity of the system [44]. It is also suggested that rare alleles are not detected if they comprise less than 4% of the PCR products [45]. To detect rare alleles and more accurately characterise a certain accession, multiple small-bulk samples or multiple single individuals from an accession should be genotyped [46]. From this perspective, the bulk sampling strategy should not be treated as a counterpart of a genotyping strategy using individuals but rather as a complementary method for the genotypic characterisation of highly heterogeneous material, such as *L. perenne*.

### Diversity and structural patterns within the *L. perenne* germplasm

Molecular markers revealed a high polymorphism rate in the examined germplasm set: for DArTs, SNPs and SSRs, 1.99, 2.00 and 8.19 alleles per locus on average were obtained across Set I. The JD for DArT markers ranged from 0.00 to 0.73; the MRD for SNPs ranged from 0.03 to 0.52; the MRD for SSR markers ranged from 0.26 to 0.76. The distribution of the genetic distance estimates was bell-shaped and similar for all marker types (Fig. 1). The allelic polymorphism and wide range of genetic distances for each marker type indicate a high level of genetic variation in the germplasm collection.

Gene diversity for dominant DArT markers was found to be 0.26; for co-dominant SNP and SSR markers it was 0.32 and 0.45, respectively. Hu et al. [18] observed a gene diversity of 0.28 within 75 *L. perenne* accessions collected from 27 countries and four continents using dominant ISSR markers, which is similar to what we found for dominant DArT markers. Brazauskas et al. [22] observed a gene diversity of 0.63 employing SSR markers on 37 European *L. perenne* accessions (380 individual genotypes), which is higher than what we obtained using SSR markers. This finding might be attributed to the abovementioned difficulty of detecting rare alleles in bulk samples and the different types of variation explored in our study. It has been well documented that in *L. perenne*, greater variation lies within the accessions than between the accessions [15, 16, 20]. Consequently, the discriminative power as well as distinction between accessions is likely to be reduced [42] because the number of common alleles is likely to increase [15].

We further subdivided the germplasm set according to the corresponding passport data and compared unique alleles, average alleles per locus and gene diversity for each subgroup (Table 3). Concerning geographical origin, Western and Northern Europe germplasm exhibited higher diversity than the other regions. However, it is difficult to draw a clear conclusion because the reduced diversity was coupled with a lower number of accessions in the germplasm set for Eastern Europe (ten accessions), Southern Europe (five accessions) and Oceania (five accessions). It is known that for highly heterogeneous material the number of samples is a significant factor in the determination of diversity [22]. Considering ploidy levels, no distinction in terms of gene diversity could be made between diploids and tetraploids, which might reflect the relationship between diploid and tetraploid *L. perenne*, since the modern tetraploid material was derived from diploid material by chemical treatment [3]. In a study on both 2× and 4× material from the same gene pool, a lack of distinction between ploidy levels was also found by Roldàn-Ruiz et al. [14]. Our finding confirms this in a broader range of material. Not surprisingly, with respect to biological status, varieties were found to possess lower diversity than breeding material or ecotypes. Successive selections have to be conducted in breeding programs to meet DUS (Distinctness, uniformity, stability) criteria. During this process, a certain number of alleles are fixed and this might reduce the available diversity [20]. The genebank material included here did not add extra diversity, which might on the one hand prove the effective maintenance of the diversity by breeders [20] or, on the other hand, provide evidence for the intensive usage of ecotypes in practical breeding work [3].

Finally we conducted STRUCTURE, PCo-based clustering and AMOVA to inspect the potential structures in Set I. In STRUCTURE analysis based on DArT markers, although the ad hoc criteria suggested eight subgroups in Set I, none of the categories defined in Table 1 could reasonably separate different clusters (Additional file 2: Figure S2). In PCo-based clustering analysis, DArT and SNP markers identified four and three subgroups when more than 65 PCos and 64 PCos were incorporated in the model; but the majority of the material was assigned to only one group (Additional file 2: Figure S3). For SSRs, the potential group number was estimated to be one after including more than 11 PCos in the model (Additional file 2: Figure S3 (a) (d)), suggesting no population structure. AMOVA analysis provided conflicting results among different marker types. For example, donor explained 3.83% of the total variance, which is the highest among all the factors for DArTs. However, ploidy level (6.39% of the total variance) and geographical origin (2.60% of the total variance) were the most influential factor for SNPs and SSRs, respectively. Despite the inconsistency, none of the factors could explain the variation within Set I to a large extent even though the variance components for all the factors were significant at *p* = 0.01.

Based on these results, there is little evidence supporting the existence of a strong structure in the tested European germplasm. In an analysis of a subset of 80 accessions of *L. perenne*, Calsyn et al. [47] found similar results i.e. geographical origins could only explain 3% of the total variation. In an association study with relatively limited or highly selected germplasm resources, a stronger structure based on origin might be identifiable [48, 49], but it is generally not expected due to the intensive usage of natural resources in breeding [20, 22] and the lack of maintenance of heterotic pools [50]. Our finding is in agreement with these statements.

### Comparison of marker systems

The application of different marker types to the same germplasm set provides opportunities to compare the performance of the marker types for diversity studies. In the present study, the estimated gene diversity was highest using SSRs, followed by SNPs and DArTs. The discrepancies should be attributable to the inherent nature of the markers and the methods used to calculate the diversity. DArT and SNP markers in the present study could be gene-associated, therefore they should be less variable in comparison to SSRs that are mainly located in non-coding regions [51]. Furthermore, Nei’s diversity measurements would favour a multi-allelic marker system like SSRs over the bi-allelic SNP and dominant DArT marker types. This was also confirmed by Van Inghelandt et al. [13] and Simko et al. [52] in their gene diversity study with different marker types. For multi-allelic marker types like SSRs, the maximum diversity value could approach one if loci are highly polymorphic; for a bi-allelic marker system like SNPs, the theoretical maximal value of this measurement could be only 0.75 in the case of an equal share of both alleles.

Unlike crops in which the pedigree information or the prior population structure can be inferred, a solid reference that could be used to compare the accuracy of accession assignment is not available in the present study. Therefore we defined two indirect criteria for the comparison: 1) the reproducibility to provide constant results for the replicated samples in Set II; 2) the consistency to provide similar results when only subsets of the data are used in a bootstrapping process.

Owing to the elimination of sampling effect, replicated samples could reveal the intrinsic reproducibility of the different marker systems. Here, we observed a high consistency for DArT markers. Within the seven replicated samples, an average JD of 0.04 was obtained and around 99% of the variation could be explained by the accessions indicating excellent reproducibility and rather low systematic error (Fig. 1, Additional file 1: Table S3). SSRs performed worst (average MRD 0.35 and only half of the variance can be explained) and SNPs performed moderately well (average MRD of 0.16 and around 70% explainable variance; Fig. 1, Additional file 1: Table S3). These findings might help to explain the lower correlations between SSRs and the other two marker types. Although DArT, SNP and SSR markers have all been reported as highly reproducible in many studies [12, 53], SSRs seemed to be more error prone in dealing with bulk samples. In the present study, the SSRs were scored manually and stutter peaks in the banding profile were commonly observed [54], which might give rise to the occurrence of higher error rates in comparison with DArTs and SNPs, especially when multiple alleles were found for a certain marker locus. In addition, it was unknown how many alleles could be expected at the different loci. The lower reproducibility of SNPs in comparison to DArTs could also be an effect of a PCR bias possibly introduced when inferring allelic frequencies from the relative signal intensity of the slide reader in combination with lower reproducibility of bulk sample SNPs on the GoldenGate assay.

In the bootstrapping analysis of the entire sample set, similar patterns among marker types were observed. The CV decreased fast when the number of markers to be resampled is small. With an increase of the number of markers, the decrease in CV tends to gradually flatten. In all scenarios, DArTs performed better than SNPs while SSRs always displayed relatively lower consistency. The decrease pattern observed in this study is similar to that described by Van Inghelandt et al. [13] and Garcia et al. [55]. Above a certain threshold a further increase of the number of markers will only slightly influence genetic distance estimates. If we set the threshold to a CV of 5% as the acceptable precision for genetic distance estimation, 554 DArT markers (40%), 110 SNP markers (60%) and 36 SSR markers (75%) are required. The ratio between SNPs and SSRs (about 3:1) in our study is much lower than the ratio of seven to eleven times more SNPs than SSRs proposed by Van Inghelandt et al. [13], in a study on maize inbred lines with 8244 SNPs and 359 SSRs. In a diversity study on sugar beet, Simko et al. [52] suggested a ratio of 4.9–13.3 between DArTs and SSRs which is lower than the one obtained in our study. Apart from the genetic differences in different crops, it appears that in dealing with bulked samples, a higher number of DArT markers is required to compensate for the loss of information due to their dominant nature. SNPs might provide more accurate estimations of allelic frequencies and therefore the information content of SNPs is likely increased. Nevertheless, DArTs in the present study outperformed the other two marker types in CV simulations owing to the immense number of markers used.

Despite the discrepancies among marker types, we have to stress that they differ mainly quantitatively, not qualitatively in this study. All of the marker systems provided similar evidence about the germplasm collections: a certain amount of diversity and polymorphism, a lack of structure and the ability to distinguish accessions. Genetic distance estimates generated by different marker types are also significantly correlated, with a high correlation coefficient. However, due to the higher consistency, better coverage of the genome and low technical dependence on prior knowledge of the sequences, DArT markers appeared to be better suited to deal with diversity studies using bulked sampling in *L. perenne.* However, with the advent of the `Genotyping by Sequencing’ techniques, Byrne et al. [25] were able to demonstrate that Genome-wide allele frequency fingerprints (GWAFF) can account for allele frequencies in bulks by read counting. In potential follow-up genotyping studies, it would be very interesting to compare the performance of GWAFF against DArTs, SSRs and SNPs in our *L. perenne* populations.

## Conclusions

Using a bulk sampling strategy, a high level of genetic diversity was found within the germplasm set in the present study. However, based on clustering analyses as well as AMOVA using passport data, a clear structure within the germplasm set was not found. All three marker types showed the capability for use in diversity studies, but DArTs showed the highest reproducibility and consistency. In addition, a subset of markers seems to be already adequate to provide reliable estimation of the genetic distances among different accessions. Based on the genetic distances and variations found within the 297 bulked accessions, the diversity study could be extended using more genotypes from certain selected accessions of interest, because there is still a large degree of diversity harboured within each accession that cannot be detected by bulk sampling. In addition, the correlation between genetic distance and heterosis could be tested in further studies and this will provide information about the potential usage of genetic distance information in breeding programs.

## Additional files

Additional file 1: Table S1. Passport data (accession name, origin, biological status, donor, ploidy level, sampling) of the examined germplasm containing 297 *Lolium perenne* L. accessions. **Table S2.** AMOVA for 297 *Lolium perenne* accessions based on genetic distance estimates using DArT, SNP and SSR markers. Source of variation was classified by geographical origin, biological status, ploidy level and donor. **Table S3.** AMOVA for the re-genotyped material based on genetic distance estimates using DArT, SNP and SSR markers. (PDF 326 kb)

Additional file 2: Figure S1. PCo results of Set III using (a) SNP, (b) SSR markers. **Figure S2**. Results of the STRUCTURE clustering for Set I containing 297 *L. perenne* accessions based on DArT markers. (a) ΔK plot for differing numbers of subpopulations (K) within the population. (b)-(e) Membership probability of assignment for Set I, subgroup number K = 8 grouped by passport information: (b) Geographical Origin; (c) Ploidy Level; (d) Biological Status; (e) Donor. **Figure S3**. (a) Number of clusters identified with mclust model with increasing number of PCo based on DArT, SNP and SSR markers; Barplot showing number of accessions in each group identified by PCo-based clustering for (b) DArTs (c) SNPs, (d) SSRs. (PPTX 337 kb)

## Abbreviations

AFLP: Amplified Fragment Length Polymorphism
AMOVA: Analysis of Molecular Variance
CV: Coefficient of Variation
DArT: Diversity Array Technology
DSV: Deutsche Saatveredelung KG
GBS: Genotyping-by-Sequencing
GD: Genetic distance
GWAFF: Genome Wide Allele Frequency Fingerprints
IPK: Leibniz Institute of Plant Genetics and Crop Plant Research
ISSR: Inter-Simple Sequence Repeat
JD: Jaccard Distance
MRD: Modified Roger’s distance
NPZ: Norddeutsche Pflanzenzucht Hans-Georg Lembke KG
PCo: Principal Coordinate
PCoA: Principal Coordinate Analysis
PIC: Polymorphic Information Content
RAPD: Random Amplification of Polymorphic DNA
SNP: Single Nucleotide Polymorphism
SSR: Single Sequence Repeat
SZS: Saatzucht Steinach GmbH & Co KG
UPGMA: Unweighted Pair Group Method with Arithmetic means
