# Supplementary material for: DArT, SNP, and SSR analyses of genetic diversity in Lolium perenne L. using bulk sampling
Source: BMC Genet. 2018 Jan 22;19:10. doi: 10.1186/s12863-017-0589-0 (PMC5778656; doi:10.1186/s12863-017-0589-0)
Supplement: Supplementary file 1 — Passport data (accession name, origin, biological status, donor, ploidy level, sampling) of the examined germplasm containing 297 Lolium perenne L. accessions. Table S2. AMOVA for 297 Lolium perenne accessions based on genetic distance estimates using DArT, SNP and SSR markers. Source of variation was classified by geographical origin, biological status, ploidy level and donor. Table S3. AMOVA for the re-genotyped material based on genetic distance estimates using DArT, SNP and SSR markers. (PDF 326 kb) [file 12863_2017_589_MOESM1_ESM.pdf]

Additional File 1: Tables

Table S1: Passport data (accession name, origin, biological status, donor, ploidy level, sampling) of the examined germplasm containing 297 *Lolium perenne* L. accessions.

Accession Name	Country of Origin	Geographic Origin	Biological Status	Donor	Ploidy	Sampling
Alligator	CHE	Western EU	variety	*standard*	4x	Population
Aubisque	NLD	Western EU	variety	*standard*	4x	Population
Argoal	FRA	Western EU	variety	*standard*	2x	Population
Delphin	GBR	Northern EU	variety	*standard*	4x	Population
Lipresso	DEU	Western EU	variety	*standard*	2x	Population
Fennema	DEU	Western EU	variety	*standard*	2x	Population
2040391	DEU	Western EU	breeding material	DSV	2x	Population
2040832	DEU	Western EU	breeding material	DSV	2x	Population
2040392	DEU	Western EU	breeding material	DSV	2x	Population
2040472	DEU	Western EU	breeding material	DSV	2x	Population
2040371	DEU	Western EU	breeding material	DSV	2x	Population
2030038	DEU	Western EU	breeding material	DSV	2x	Population
2030270	DEU	Western EU	breeding material	DSV	2x	Population
6010	NLD	Western EU	breeding material	DSV	2x	Population
6015	NLD	Western EU	breeding material	DSV	2x	Population
6017	NLD	Western EU	breeding material	DSV	2x	Population
6018	NLD	Western EU	breeding material	DSV	2x	Population
6023	NLD	Western EU	breeding material	DSV	2x	Population
6027	NLD	Western EU	breeding material	DSV	2x	Population
6030	NLD	Western EU	breeding material	DSV	2x	Population
6032	NLD	Western EU	breeding material	DSV	2x	Population
6033	NLD	Western EU	breeding material	DSV	2x	Population
6034	NLD	Western EU	breeding material	DSV	2x	Population
6045	NLD	Western EU	breeding material	DSV	2x	Population
6046	NLD	Western EU	breeding material	DSV	2x	Population
6048	NLD	Western EU	breeding material	DSV	2x	Population
6050	NLD	Western EU	breeding material	DSV	2x	Population
6051	NLD	Western EU	breeding material	DSV	2x	Population
6052	NLD	Western EU	breeding material	DSV	2x	Population
6055	NLD	Western EU	breeding material	DSV	4x	Population
6057	NLD	Western EU	breeding material	DSV	4x	Population
6058	NLD	Western EU	breeding material	DSV	4x	Population
6062	NLD	Western EU	breeding material	DSV	4x	Population
6064	NLD	Western EU	breeding material	DSV	4x	Population
6066	NLD	Western EU	breeding material	DSV	4x	Population
6068	NLD	Western EU	breeding material	DSV	4x	Population
6070	NLD	Western EU	breeding material	DSV	4x	Population
6071	NLD	Western EU	breeding material	DSV	4x	Population

6072	NLD	Western EU	breeding material	DSV	4x	Population
6073	NLD	Western EU	breeding material	DSV	4x	Population
ASTURION	NLD	Western EU	variety	DSV	2x	Population
ASTORGA	NLD	Western EU	variety	DSV	2x	Population
ZLp_96-024	NLD	Western EU	unknown	DSV	2x	Population
ASMIR	FRA	Western EU	variety	DSV	4x	Population
LIMBOS	NLD	Western EU	variety	DSV	4x	Population
OCTAVIO	NLD	Western EU	variety	DSV	2x	Population
VAUDAIRE	DEU	Western EU	variety	DSV	2x	Population
SURES	DEU	Western EU	variety	DSV	4x	Population
ASTONENERGY	GBR	Northern EU	variety	DSV	4x	Population
Lp_9928D	NZL	Oceania	unknown	DSV	2x	Population
Pop_Bn	NZL	Oceania	variety	DSV	2x	Population
Pop_Q	NZL	Oceania	variety	DSV	2x	Population
KRC_6625	NZL	Oceania	unknown	DSV	2x	Population
KRC_6626	NZL	Oceania	unknown	DSV	2x	Population
SLp092046	IRL	Northern EU	ecotype	DSV	2x	Population
SLp092052	IRL	Northern EU	ecotype	DSV	2x	Population
SLp092044	IRL	Northern EU	ecotype	DSV	2x	Population
SLp092048	IRL	Northern EU	ecotype	DSV	2x	Population
SLp092039	IRL	Northern EU	ecotype	DSV	2x	Population
SLp092003	IRL	Northern EU	ecotype	DSV	2x	Population
SLp092050	IRL	Northern EU	ecotype	DSV	2x	Population
SLp092017	IRL	Northern EU	ecotype	DSV	2x	Population
SLp_080901	IRL	Northern EU	ecotype	DSV	2x	Population
CMS_236_A	unknown	unknown	breeding material	NPZ	2x	Population
CMS_213_A	unknown	unknown	breeding material	NPZ	2x	Population
CMS_237_A	unknown	unknown	breeding material	NPZ	2x	Population
Inoval	FRA	Western EU	variety	NPZ	2x	Population
Urspr.klon_CMS_206	unknown	unknown	breeding material	NPZ	2x	Clone
Urspr.klon_CMS_213	unknown	unknown	breeding material	NPZ	2x	Clone
Urspr.klon_CMS_215	NLD	Western EU	breeding material	NPZ	2x	Clone
Urspr.klon_CMS_216	NLD	Western EU	breeding material	NPZ	2x	Clone
Urspr.klon_CMS_236	unknown	unknown	breeding material	NPZ	2x	Clone
Urspr.klon_CMS_237	NLD	Western EU	breeding material	NPZ	2x	Clone
Urspr.klon_CMS_238	NLD	Western EU	breeding material	NPZ	2x	Clone
Urspr.klon_CMS_240	unknown	unknown	breeding material	NPZ	2x	Clone
Urspr.klon_CMS_246	unknown	unknown	breeding material	NPZ	2x	Clone
Maint._MSF1_403	NLD	Western EU	breeding material	NPZ	2x	Clone
Maint._MSF1_459	NLD	Western EU	breeding material	NPZ	2x	Clone
Maint._MSF1_460	unknown	unknown	breeding material	NPZ	2x	Clone
Best._MSF1_451	unknown	unknown	breeding material	NPZ	2x	Clone
Best._MSF1_461	unknown	unknown	breeding material	NPZ	2x	Clone
Best._MSF1_500	NLD	Western EU	breeding material	NPZ	2x	Clone
Best._MSF1_502	GBR	Northern EU	breeding material	NPZ	2x	Clone
Best._MSF1_503	NLD	Western EU	breeding material	NPZ	2x	Clone

Best._MSF1_504	NLD	Western EU	breeding material	NPZ	2x	Clone
Best._MSF1_507	FRA	Western EU	breeding material	NPZ	2x	Clone
Best._MSF1_508	FRA	Western EU	breeding material	NPZ	2x	Clone
Best._MSF1_509	GBR	Northern EU	breeding material	NPZ	2x	Clone
Best._MSF1_510	unknown	unknown	breeding material	NPZ	2x	Clone
Best._MSF1_511	FRA	Western EU	breeding material	NPZ	2x	Clone
PC_08_2805	NLD	Western EU	breeding material	NPZ	2x	Clone
PC_08_2808	unknown	unknown	breeding material	NPZ	2x	Clone
PC_08_2902	ITA	Southern EU	ecotype	NPZ	2x	Clone
PC_08_3006	HUN	Eastern EU	ecotype	NPZ	2x	Clone
PC_08_3008	GRC	Southern EU	ecotype	NPZ	2x	Clone
PC_09_2902	unknown	unknown	breeding material	NPZ	2x	Clone
PC_09_2903	DNK	Northern EU	breeding material	NPZ	2x	Clone
PC_09_2908	unknown	unknown	breeding material	NPZ	2x	Clone
Aberavon	GBR	Northern EU	variety	NPZ	2x	Population
Pop_A	NLD	Western EU	variety	NPZ	2x	Population
Urspr.klon_CMS_217	unknown	unknown	breeding material	NPZ	4x	Clone
Urspr.klon_CMS_221	FRA	Western EU	breeding material	NPZ	4x	Clone
Urspr.klon_CMS_225	unknown	unknown	breeding material	NPZ	4x	Clone
Urspr.klon_CMS_228	NLD	Western EU	breeding material	NPZ	4x	Clone
Urspr.klon_CMS_229	unknown	unknown	breeding material	NPZ	4x	Clone
Urspr.klon_CMS_230	DNK	Northern EU	breeding material	NPZ	4x	Clone
Urspr.klon_CMS_231	FRA	Western EU	breeding material	NPZ	4x	Clone
Urspr.klon_CMS_232	unknown	unknown	breeding material	NPZ	4x	Clone
Urspr.klon_CMS_233	NLD	Western EU	breeding material	NPZ	4x	Clone
Urspr.klon_CMS_234	DNK	Northern EU	breeding material	NPZ	4x	Clone
Urspr.klon_CMS_235	DNK	Northern EU	breeding material	NPZ	4x	Clone
Urspr.klon_CMS_239	BEL	Western EU	breeding material	NPZ	4x	Clone
Urspr.klon_CMS_241	NLD	Western EU	breeding material	NPZ	4x	Clone
Urspr.klon_CMS_242	unknown	unknown	breeding material	NPZ	4x	Clone
Urspr.klon_CMS_243	unknown	unknown	breeding material	NPZ	4x	Clone
Urspr.klon_CMS_244	unknown	unknown	breeding material	NPZ	4x	Clone
Maint._MSF1_384	unknown	unknown	breeding material	NPZ	4x	Clone
Maint._MSF1_423	BEL	Western EU	breeding material	NPZ	4x	Clone
Best._MSF1_455	unknown	unknown	breeding material	NPZ	4x	Clone
Best._MSF1_464	unknown	unknown	breeding material	NPZ	4x	Clone
Best._MSF1_470	unknown	unknown	breeding material	NPZ	4x	Clone
Best._MSF1_471	unknown	unknown	breeding material	NPZ	4x	Clone
Best._MSF1_481	unknown	unknown	breeding material	NPZ	4x	Clone
PC_08_4801	unknown	unknown	breeding material	NPZ	4x	Clone
PC_09_4803	unknown	unknown	breeding material	NPZ	4x	Clone
PC_09_4901	unknown	unknown	breeding material	NPZ	4x	Clone
PC_09_4805	unknown	unknown	breeding material	NPZ	4x	Clone
PC_09_4902	unknown	unknown	breeding material	NPZ	4x	Clone
PC_09_4903	unknown	unknown	breeding material	NPZ	4x	Clone
PC_09_4808	DNK	Northern EU	breeding material	NPZ	4x	Clone

PC_09_4904	BEL	Western EU	breeding material	NPZ	4x	Clone
PC_09_4906	NLD	Western EU	breeding material	NPZ	4x	Clone
Syn_06_4501	FRA	Western EU	breeding material	NPZ	4x	Clone
Syn_06_4503	unknown	unknown	breeding material	NPZ	4x	Clone
Syn_06_4505	unknown	unknown	breeding material	NPZ	4x	Clone
Syn_06_4702	unknown	unknown	breeding material	NPZ	4x	Clone
Syn_08_4001	unknown	unknown	breeding material	NPZ	4x	Clone
Syn_08_4103	unknown	unknown	breeding material	NPZ	4x	Clone
Syn_08_4408	GBR	Northern EU	breeding material	NPZ	4x	Clone
Syn_08_4507	GBR	Northern EU	breeding material	NPZ	4x	Clone
Syn_08_4605	FRA	Western EU	breeding material	NPZ	4x	Clone
Syn_08_4606	FRA	Western EU	breeding material	NPZ	4x	Clone
Syn_08_4607	unknown	unknown	breeding material	NPZ	4x	Clone
NPZ_FNR_1_(2010)	unknown	unknown	breeding material	NPZ	4x	Population
2060005	DEU	Western EU	breeding material	DSV	2x	Population
2060030	DEU	Western EU	breeding material	DSV	2x	Population
2060166	DEU	Western EU	breeding material	DSV	2x	Population
2060452	DEU	Western EU	breeding material	DSV	2x	Population
2060912	DEU	Western EU	breeding material	DSV	2x	Population
2060956	DEU	Western EU	breeding material	DSV	2x	Population
2062148	DEU	Western EU	breeding material	DSV	2x	Population
2062153	DEU	Western EU	breeding material	DSV	2x	Population
2090502	DEU	Western EU	breeding material	DSV	2x	Population
2090505	DEU	Western EU	breeding material	DSV	2x	Population
2090516	DEU	Western EU	breeding material	DSV	2x	Population
2060033	DEU	Western EU	breeding material	DSV	2x	Population
2060049	DEU	Western EU	breeding material	DSV	2x	Population
2060072	DEU	Western EU	breeding material	DSV	2x	Population
2060118	DEU	Western EU	breeding material	DSV	2x	Population
2060123	DEU	Western EU	breeding material	DSV	2x	Population
2060356	DEU	Western EU	breeding material	DSV	2x	Population
2060480	DEU	Western EU	breeding material	DSV	2x	Population
2060497	DEU	Western EU	breeding material	DSV	2x	Population
2060927	DEU	Western EU	breeding material	DSV	2x	Population
2090503	DEU	Western EU	breeding material	DSV	2x	Population
2090504	DEU	Western EU	breeding material	DSV	2x	Population
2083007	DEU	Western EU	breeding material	DSV	2x	Population
2083010	DEU	Western EU	breeding material	DSV	2x	Population
2060286	DEU	Western EU	breeding material	DSV	2x	Population
2060328	DEU	Western EU	breeding material	DSV	2x	Population
2060756	DEU	Western EU	breeding material	DSV	2x	Population
2060900	DEU	Western EU	breeding material	DSV	2x	Population
2060903	DEU	Western EU	breeding material	DSV	2x	Population
2062031	DEU	Western EU	breeding material	DSV	2x	Population
2062060	DEU	Western EU	breeding material	DSV	2x	Population
2062166	DEU	Western EU	breeding material	DSV	2x	Population

91623	NLD	Western EU	breeding material	DSV	2x	Population
2030872	DEU	Western EU	breeding material	DSV	2x	Population
2030323	DEU	Western EU	breeding material	DSV	2x	Population
2030350	DEU	Western EU	breeding material	DSV	2x	Population
2030367	DEU	Western EU	breeding material	DSV	2x	Population
2020548	DEU	Western EU	breeding material	DSV	2x	Population
2030830	DEU	Western EU	breeding material	DSV	2x	Population
2030926	DEU	Western EU	breeding material	DSV	2x	Population
2030337	DEU	Western EU	breeding material	DSV	2x	Population
2030117	DEU	Western EU	breeding material	DSV	2x	Population
2020795	DEU	Western EU	breeding material	DSV	2x	Population
106232	DEU	Western EU	breeding material	DSV	2x	Population
2040121	DEU	Western EU	breeding material	DSV	2x	Population
2030377	DEU	Western EU	breeding material	DSV	2x	Population
2040371	DEU	Western EU	breeding material	DSV	2x	Population
S4	FRA	Western EU	variety	SZS	2x	Population
S11	DEU	Western EU	breeding material	SZS	2x	Population
S12	DEU	Western EU	breeding material	SZS	2x	Population
S14	DEU	Western EU	breeding material	SZS	2x	Population
S15	DEU	Western EU	breeding material	SZS	2x	Population
S21	DEU	Western EU	breeding material	SZS	2x	Population
S22	DEU	Western EU	breeding material	SZS	2x	Population
S23	DEU	Western EU	breeding material	SZS	2x	Population
S26	DEU	Western EU	breeding material	SZS	2x	Population
S35	DEU	Western EU	breeding material	SZS	2x	Population
S40	DEU	Western EU	breeding material	SZS	2x	Population
40544	DEU	Western EU	breeding material	SZS	2x	Population
40575	DEU	Western EU	breeding material	SZS	2x	Population
40603	DEU	Western EU	breeding material	SZS	2x	Population
40634	DEU	Western EU	breeding material	SZS	2x	Population
40664	DEU	Western EU	breeding material	SZS	2x	Population
40695	DEU	Western EU	breeding material	SZS	2x	Population
GR3109	DNK	Northern EU	variety	IPK	2x	Population
GR5041	DEU	Western EU	ecotype	IPK	2x	Population
GR8422	FRA	Western EU	ecotype	IPK	2x	Population
GR8428	GBR	Northern EU	breeding material	IPK	2x	Population
GR3091	DNK	Northern EU	variety	IPK	2x	Population
GR3525	DEU	Western EU	ecotype	IPK	2x	Population
GR7867	NLD	Western EU	variety	IPK	2x	Population
GR8419	NLD	Western EU	variety	IPK	2x	Population
GR3107	GBR	Northern EU	variety	IPK	2x	Population
GR3231	FIN	Northern EU	variety	IPK	2x	Population
GR3368	POL	Eastern EU	ecotype	IPK	2x	Population
GR3511	CZE	Eastern EU	ecotype	IPK	2x	Population
GR2704	DNK	Northern EU	variety	IPK	2x	Population
GR2725	GBR	Northern EU	variety	IPK	2x	Population

GR2910	FIN	Northern EU	breeding material	IPK	2x	Population
GR3084	FRA	Western EU	variety	IPK	2x	Population
GR3236	BEL	Western EU	variety	IPK	2x	Population
GR3243	NLD	Western EU	variety	IPK	2x	Population
GR3352	DEU	Western EU	ecotype	IPK	2x	Population
GR2859	DEU	Western EU	ecotype	IPK	2x	Population
GR2915	GBR	Northern EU	landrace	IPK	2x	Population
GR2929	RUS	Eastern EU	breeding material	IPK	2x	Population
GR3122	RUS	Eastern EU	landrace	IPK	2x	Population
GR3467	DEU	Western EU	ecotype	IPK	2x	Population
GR5646	unknown	unknown	ecotype	IPK	2x	Population
GR6882	ESP	Southern EU	ecotype	IPK	4x	Population
GR8420	FRA	Western EU	ecotype	IPK	2x	Population
GR3142	CSK	Eastern EU	variety	IPK	2x	Population
GR3172	POL	Eastern EU	variety	IPK	4x	Population
GR3373	POL	Eastern EU	ecotype	IPK	2x	Population
GR3550	CZE	Eastern EU	ecotype	IPK	2x	Population
GR5015	DNK	Northern EU	variety	IPK	2x	Population
GR5100	DEU	Western EU	ecotype	IPK	2x	Population
GR5112	DEU	Western EU	ecotype	IPK	2x	Population
GR5113	DEU	Western EU	ecotype	IPK	2x	Population
GR7398	ESP	Southern EU	ecotype	IPK	2x	Population
GR7420	ESP	Southern EU	ecotype	IPK	2x	Population
GR7672	SWE	Northern EU	variety	IPK	2x	Population
GR7804	IRL	Northern EU	ecotype	IPK	2x	Population
GR8340	SWE	Northern EU	variety	IPK	2x	Population
GR8502	IRL	Northern EU	variety	IPK	2x	Population
GR8605	FRA	Western EU	ecotype	IPK	2x	Population
GR8611	FRA	Western EU	ecotype	IPK	2x	Population
GR8808	NLD	Western EU	variety	IPK	2x	Population
GR8826	ROM	Eastern EU	ecotype	IPK	2x	Population
GR9013	FIN	Northern EU	breeding material	IPK	2x	Population
GR6598	unknown	unknown	ecotype	IPK	2x	Population
GR9047	NLD	Western EU	variety	IPK	2x	Population
SH_2/74	DEU	Western EU	ecotype	NPZ	2x	Population
SH_3/109	DEU	Western EU	ecotype	NPZ	2x	Population
SH_3/128	DEU	Western EU	ecotype	NPZ	2x	Population
SH_4/155	DEU	Western EU	ecotype	NPZ	2x	Population
SH_5/181	DEU	Western EU	ecotype	NPZ	2x	Population
SH_5/187	DEU	Western EU	ecotype	NPZ	2x	Population
SH_6/222	DEU	Western EU	ecotype	NPZ	2x	Population
SH_7/282	DEU	Western EU	ecotype	NPZ	2x	Population
Pop_BR	NLD	Western EU	variety	NPZ	4x	Population
107802	DEU	Western EU	breeding material	DSV	2x	Population
2020271	DEU	Western EU	breeding material	DSV	2x	Population
2020340	DEU	Western EU	breeding material	DSV	2x	Population

2020432	DEU	Western EU	breeding material	DSV	2x	Population
2020505	DEU	Western EU	breeding material	DSV	2x	Population
2020511	DEU	Western EU	breeding material	DSV	2x	Population
2020662	DEU	Western EU	breeding material	DSV	2x	Population
2030083	DEU	Western EU	breeding material	DSV	2x	Population
2030176	DEU	Western EU	breeding material	DSV	2x	Population
2030331	DEU	Western EU	breeding material	DSV	2x	Population
2030359	DEU	Western EU	breeding material	DSV	2x	Population
2030406	DEU	Western EU	breeding material	DSV	2x	Population
2030442	DEU	Western EU	breeding material	DSV	2x	Population
2030472	DEU	Western EU	breeding material	DSV	2x	Population
2030529	DEU	Western EU	breeding material	DSV	2x	Population
2030611	DEU	Western EU	breeding material	DSV	2x	Population
2030721	DEU	Western EU	breeding material	DSV	2x	Population
2030783	DEU	Western EU	breeding material	DSV	2x	Population
2030867	DEU	Western EU	breeding material	DSV	2x	Population
2040102	DEU	Western EU	breeding material	DSV	2x	Population
2040352	DEU	Western EU	breeding material	DSV	2x	Population
2040606	DEU	Western EU	breeding material	DSV	2x	Population
40725	DEU	Western EU	breeding material	SZS	2x	Population
40756	DEU	Western EU	breeding material	SZS	2x	Population
40787	DEU	Western EU	breeding material	SZS	2x	Population
40817	DEU	Western EU	breeding material	SZS	2x	Population
40848	DEU	Western EU	breeding material	SZS	2x	Population
2011-12_1	DEU	Western EU	breeding material	SZS	2x	Population
2011-13_1	FRA	Western EU	variety	SZS	2x	Population
2011-14_1	DEU	Western EU	breeding material	SZS	2x	Population
2011-15_1	DEU	Western EU	breeding material	SZS	2x	Population
2011-16_1	GBR	Northern EU	variety	SZS	2x	Population

Table S2: AMOVA for 297 *Lolium perenne* accessions based on genetic distance estimates using DArT, SNP and SSR markers. Source of variation was classified by geographical origin, biological status, ploidy level and donor.

Source of Variation	df	DArT			SNP			SSR		
		Sum of squares	Variance Component[c]	Variance%	Sum of squares	Variance Component[c]	Variance%	Sum of squares	Variance Component[c]	Variance%
Geographical origin[a]										
Among Geographical origin groups	4	0.640	0.003	2.64%	0.289	0.001	1.36%	0.928	0.004	2.60%
Within Geographical origin groups	249	24.210	0.097	97.36%	13.530	0.054	98.64%	35.306	0.142	97.40%
Biological Status										
Among Biological status groups	4	0.820	0.003	2.81%	0.353	0.001	1.49%	0.974	0.003	1.88%
Within Biological status	292	29.575	0.101	97.19%	16.773	0.057	98.51%	42.300	0.145	98.12%
Ploidy level										
Among Ploidy groups	1	0.396	0.003	2.76%	0.449	0.004	6.39%	0.442	0.003	1.97%
Within Ploidy groups	295	30.000	0.102	97.23%	16.678	0.057	93.61%	42.831	0.145	98.03%
Donor [b]										
Among Donor groups	3	1.101	0.004	3.83%	0.394	0.001	1.88%	1.171	0.004	2.51%
Within Donor groups	287	29.004	0.101	96.17%	16.582	0.058	98.11%	41.440	0.144	97.49%

[a] material with unknown origin was removed

[b] standard cultivar was removed

[c] variance component was all significant at $p = 0.01$ after 1000 permutation.

Table S3. AMOVA for the re-genotyped material based on genetic distance estimates using DArT, SNP and SSR markers

	Source of Variation	df	Variance Component[a]	Variance%
	Among replicates groups	5	0.086	98.96
**DArT**	Within replicates groups	7	0.001	1.04
	Total	12		
	Among replicates groups	5	0.033	69.95
**SNP**	Within replicates groups	7	0.014	30.05
	Total	12		
	Among replicates groups	4	0.077	52.32
**SSR[b]**	Within replicates groups[b]	6	0.070	47.68
	Total	10		

[a] all of the variance components were significant at p = 0.01 after 1000 permutations.

[b] for SSRs, one sample was excluded due to a high missing value rate.
