# Supplementary material for: DArT, SNP, and SSR analyses of genetic diversity in Lolium perenne L. using bulk sampling
Source: BMC Genet. 2018 Jan 22;19:10. doi: 10.1186/s12863-017-0589-0 (PMC5778656; doi:10.1186/s12863-017-0589-0)
Supplement: Supplementary file 2 — PCo results of Set III using (a) SNP, (b) SSR markers. Figure S2. Results of the STRUCTURE clustering for Set I containing 297 L. perenne accessions based on DArT markers. (a) ΔK plot for differing numbers of subpopulations (K) within the population. (b)-(e) Membership probability of assignment for Set I, subgroup number K = 8 grouped by passport information: (b) Geographical Origin; (c) Ploidy Level; (d) Biological Status; (e) Donor. Figure S3. (a) Number of clusters identified with mclust model with increasing number of PCo based on DArT, SNP and SSR markers; Barplot showing number of accessions in each group identified by PCo-based clustering for (b) DArTs (c) SNPs, (d) SSRs. (PPTX 337 kb) [file 12863_2017_589_MOESM2_ESM.pptx]

## Slide 1
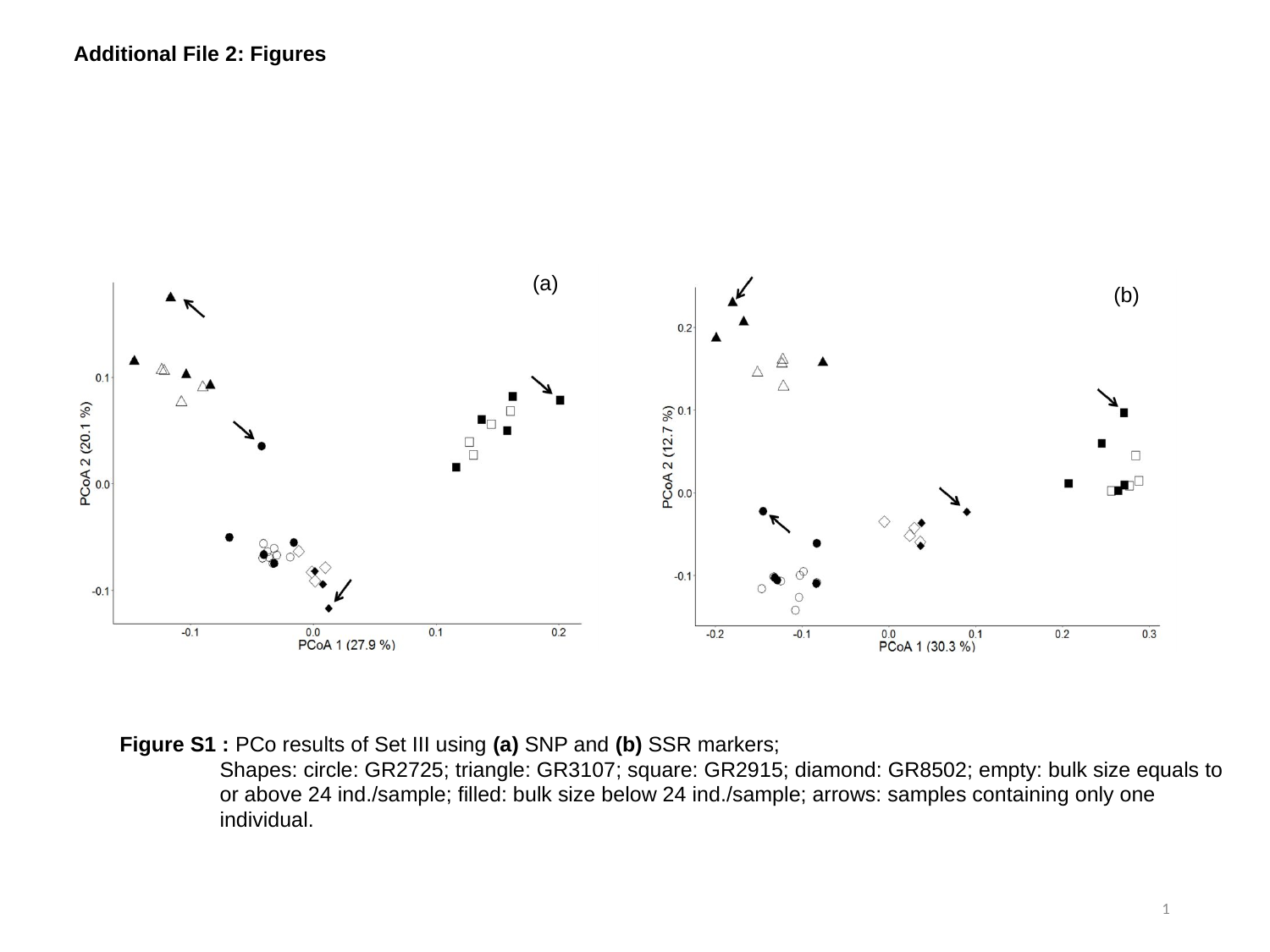

Additional File 2: Figures
(a)
(b)
Figure S1 : PCo results of Set III using (a) SNP and (b) SSR markers;
	Shapes: circle: GR2725; triangle: GR3107; square: GR2915; diamond: GR8502; empty: bulk size equals to or above 24 ind./sample; filled: bulk size below 24 ind./sample; arrows: samples containing only one individual.
1

## Slide 2
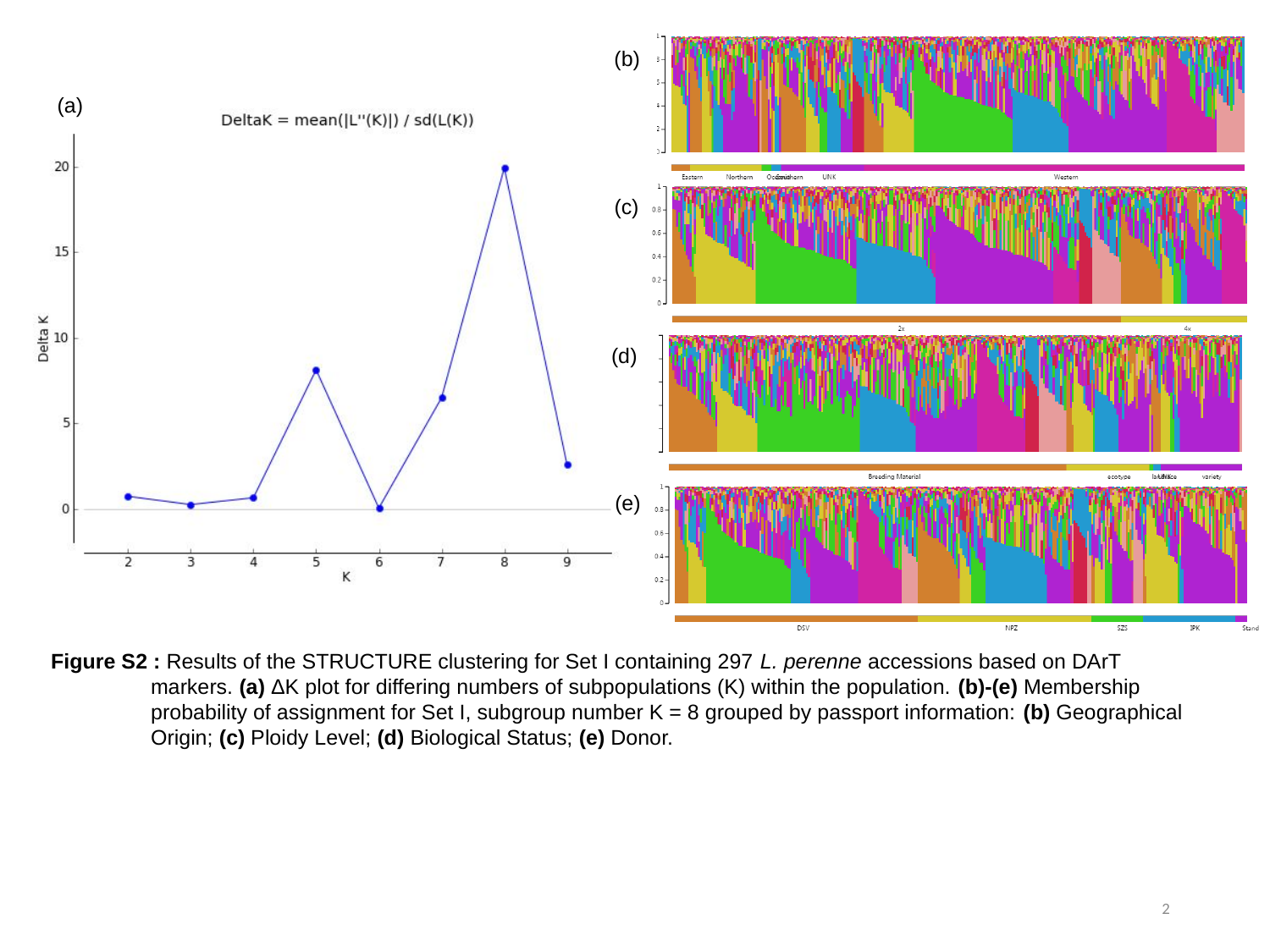

(b)
(a)
(c)
(d)
(e)
Figure S2 : Results of the STRUCTURE clustering for Set I containing 297 L. perenne accessions based on DArT markers. (a) ΔK plot for differing numbers of subpopulations (K) within the population. (b)-(e) Membership probability of assignment for Set I, subgroup number K = 8 grouped by passport information: (b) Geographical Origin; (c) Ploidy Level; (d) Biological Status; (e) Donor.
2

## Slide 3
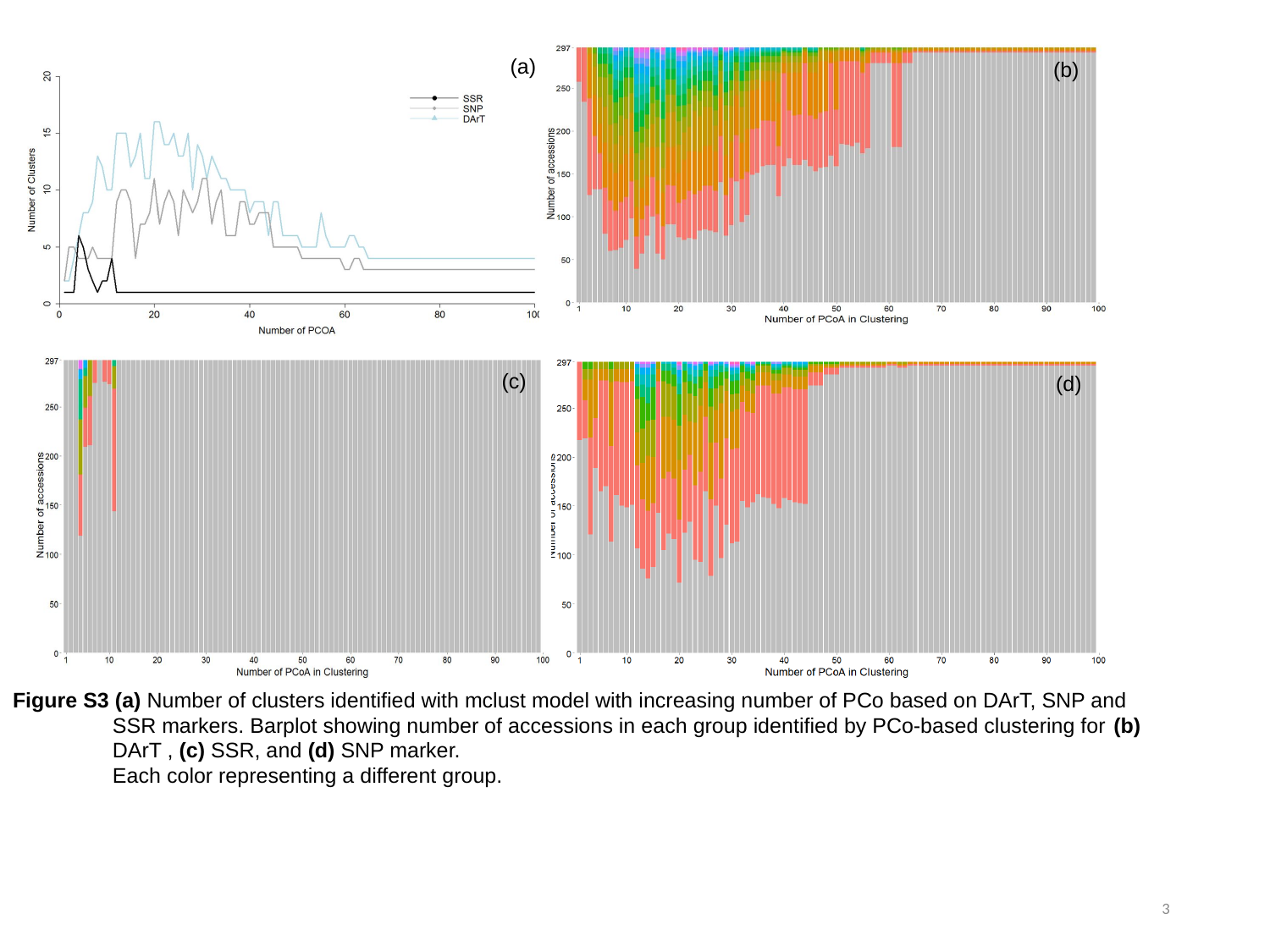

(a)
(b)
(c)
(d)
Figure S3 (a) Number of clusters identified with mclust model with increasing number of PCo based on DArT, SNP and SSR markers. Barplot showing number of accessions in each group identified by PCo-based clustering for (b) DArT , (c) SSR, and (d) SNP marker.
	Each color representing a different group.
3
